# CNN‐based deep learning approach for classification of invasive ductal and metastasis types of breast carcinoma

**DOI:** 10.1002/cam4.70069

**Published:** 2024-08-30

**Authors:** Tobibul Islam, Md Enamul Hoque, Mohammad Ullah, Toufiqul Islam, Nabila Akter Nishu, Rabiul Islam

**Affiliations:** ^1^ Department of Biomedical Engineering Military Institute of Science and Technology Dhaka Bangladesh; ^2^ Center for Advance Intelligent Materials Universiti Malaysia Pahang Kuantan Malaysia; ^3^ Department of Surgery M Abdur Rahim Medical College Dinajpur Bangladesh; ^4^ Department of Medicine Armed Forces Medical College Dhaka Bangladesh; ^5^ Department of Electrical and Computer Engineering Texas A&M University College Station Texas USA

**Keywords:** computer‐aided diagnosis (CAD), convolutional neural network, histopathological image, invasive ductal carcinoma (IDC), metastasis

## Abstract

**Objective:**

Breast cancer is one of the leading cancer causes among women worldwide. It can be classified as invasive ductal carcinoma (IDC) or metastatic cancer. Early detection of breast cancer is challenging due to the lack of early warning signs. Generally, a mammogram is recommended by specialists for screening. Existing approaches are not accurate enough for real‐time diagnostic applications and thus require better and smarter cancer diagnostic approaches. This study aims to develop a customized machine‐learning framework that will give more accurate predictions for IDC and metastasis cancer classification.

**Methods:**

This work proposes a convolutional neural network (CNN) model for classifying IDC and metastatic breast cancer. The study utilized a large‐scale dataset of microscopic histopathological images to automatically perceive a hierarchical manner of learning and understanding.

**Results:**

It is evident that using machine learning techniques significantly (15%–25%) boost the effectiveness of determining cancer vulnerability, malignancy, and demise. The results demonstrate an excellent performance ensuring an average of 95% accuracy in classifying metastatic cells against benign ones and 89% accuracy was obtained in terms of detecting IDC.

**Conclusions:**

The results suggest that the proposed model improves classification accuracy. Therefore, it could be applied effectively in classifying IDC and metastatic cancer in comparison to other state‐of‐the‐art models.

## 1 INTRODUCTION

Around one in eight US women may develop invasive breast cancer throughout their lifetime as of January 27, 2020.^1^, ^2^ Whenever any form of the disorder is diagnosed by medical image analysis, the computer‐aided diagnosis (CADx) system takes precedence. It gives pathologists a workflow as it takes only a little time to execute the results.^3^, ^4^ Cancer is defined as a heterogeneous disease made up of several diverse subgroups.^5^ Abnormal cells are not carcinogenic but can sometimes boost the risk of cancer. The non‐spreadable unusual cells are marked as noninvasive. Often this is called pre‐cancer, or level 0 cancer, for example, invasive ductal carcinoma (IDC) or ductal carcinoma in situ (DCIS).^6^ IDC is indeed a milk duct breast cancer and is not invasive. It has not progressed further than the duct from where it originated. If unusual cells travel outside the tissue barrier where they are formed, then the cells appear invasive.^7^ When these unusual cells spread out into surrounding breast tissue within the milk ducts, this is known as invasive breast cancer.^8^ Such cells may also move away and disperse to certain other body parts from the host site. Cancerous cells head for the nearby blood or lymph vessels when they grow forward.^9^ From there, the cells can migrate through the bloodstream or lymph system to meet other body parts. Once this develops, the cells develop tiny tumors called “micrometastases” at first.^10^ Such tiny tumors cause the development of new blood vessels that are then used to drive tumor growth. This form of tumor is labeled as a metastatic tumor.^11^ Metastatic cancer is more dangerous and harder to cure because it creates a web of abnormalities throughout the body and often, we can not recognize from where it originated.^12^ Metastatic cancer is normally called stage IV cancer.^13^ With early diagnosis, it is possible to shrink the tumor cells and stop the cell growth somehow which can prolong the lifetime of a cancer patient. Therefore, if metastasis is not early diagnosed, the cell growth will be more rapid and large. But it does not mean metastatic cancer is not curable. If metastatic cancers are diagnosed early, patients may recover optimally from successful early therapeutic interventions. For this form of intelligent image processing, machine learning or more specifically deep neural networks can be used with success.

Models based on CNN consist of multiple layers of convolutional and pooling layers.^14^ The convolutional layer can be considered as running sliding windows over inputs and multiplying the components.^15^ It uses matrix multiplications instead of loops since these scale better and operate faster.^16^ Compared to conventional completely connected layers, coevolutionary layers have a few parameters in which a model uses the same parameters in more than one location.^17^ That enables the model more effective, both statistically and in terms of computation. CNN has excellent feature extraction capabilities in medical images.^18^

To chase the classification of breast cancer cells, several detection approaches through image analysis have been previously explored by many researchers. Petushi et al.^19^ developed an automated computational method for tissue classification based on micro‐texture features of tissues by dividing the nuclei into segments and analyzing them, two textural features (nuclei surface density along with spatial position) were extracted. The proposed approach was employed to differentiate between different categories of tumor cells as well as surrounding tissues such as stroma or adipose tissue. The proposed method can be an efficient tool in classifying the tumor cells based upon the subdivision of the whole slide images having a dense concentration of cancer cell nuclei that aligns with the slide's overall grade classification. Unlike the paper approaching binary classification, Zhongyi et al. prioritized the multi‐classification of breast cancer.^20^ According to their proposal, a comprehensive model based on a class structure‐based deep convolutional neural network (CSDCNN) that pursued an end‐to‐end training manner and is capable of learning semantic features from low level to high level. The design of CSDCNN has been developed to accomplish an account of the relation of feature spaces among intra‐class. Here, inter‐class as well with a noteworthy performance, and accuracy of an average of 93.2% on the demonstration of multi‐classification of breast cancer cells has been achieved so.

Spaniel et al.^21^ brought out an approach that provided a map of binary classification that is designed to categorize benign and malignant tumors through different feature descriptors and machine‐learning classifiers. They drew out patches of 32 × 32 and 64 × 64 from the images to instruct their CNN. Here, the accuracy gained was around 80% to 85%. Cruz Roa et.al^22^ built up a trained CNN that operated on 100 × 100 size patches taken out. Among the 3 layers of CNN architecture, two of the layers were utilized for performing convolution and pooling operations, while the remaining layers are fully connected. Using this, they constructed a probability map where the predicted IDC regions were highlighted and perceived a balanced accuracy of about 84.23%. On an overall slide level, Bejnordi et al.^23^ approach can automatically identify ductal carcinoma in situ (DCIS) and distinguish it from the benign area. They developed a novel data‐driven system that is primarily susceptible to demonstrating stromal morphological features to distinguish between those with breast cancer and those who have benign breast ailments with an accuracy of about 92%. Beck et al.^24^ generated a C‐Path system to measure a rich number of morphological features that could determine the characteristics of prognostic relevance to provide a prompt means for assessing prognosis from microscopic image data. The significance of stromal morphological traits as a crucial prognostic factor in breast cancer was demonstrated in new ways by this model. Paper^25^ created a computer‐aided diagnosis (CAD) system that can detect and differentiate between benign and malignant tumors in breast mammography images. The system required a region of interest (ROI) and threshold‐based approaches to segment the images. DCNN framework AlexNet is being used for the extraction of the elements. The support vector machine classifier was merged with the final fully connected layer to increase accuracy. They utilized two public datasets: (i) the digital mammography screening database (DDSM), and (ii) DDSM's Curated Breast Imaging Subset (CBIS‐DDSM). In,^19^ a CAD method is built for masses in the volume of digital breast tomosynthesis (DBT) that uses a deep coevolutionary neural network (DCNN) with mammographic transfer learning. It used digitized films and mammogram images. To minimize overfitting, jittering, and dropout strategies were employed. The performance of two CAD mass detection systems in DBT was compared that used the DCNN and FP reduction feature‐based approaches.

The accuracy level of the proposed CNN model is compared with the other methods regarding breast cancer detection shown in Table 4. Dina et al. proposed a method where they used the combination of DCNN, SVM, and AlexNet methods for detecting breast cancer detection, where the accuracy level was 87.2%.^19^ In the same way, Zhongyi et al. used CSDCNN and the multiclassification method where they got 93.2% accuracy.^26^ In other words, Shweta et al. analyzed breast cancer based on the pre‐trained network extracted traits and SVM and achieved an average accuracy of 90.12%.^27^ Moreover, Saad et al. used the CNN technique to classify cancer detection by examining the zones of hostile ductal carcinoma tissue in whole‐slide images (WSIs). The success rate of that proposed system was 87%. Fabio et al. proposed a handcrafted feature‐based breast cancer classification method based on the BreaKHis dataset and achieved 94.54% accuracy.^28^ Kalpana et al. stated a method based on three training strategies: nucleus patches, transfer learning, and classifier fusion. The average level of success rate was 96.91 ± 0.67 [29n]. Long and colleagues proposed a novel deep neural network structure that uses transfer learning for the classification of microscopic images. Their proposed network utilizes the features extracted from three pre‐trained deep CNNs, where the accuracy was 92.63 ± 1.68.^29^ Using the BreaKHis dataset, Gour et al.^17^ proposed the ResHist model, a modified version of the ResNet‐152 architecture that can be utilized for breast cancer classification. For various magnification factors, one can expect an average accuracy of 91.35 ± 2.3 at the image level by using this technique. Shallu and colleagues established a framework that focuses on fine‐tuned pre‐trained VGG16 networks where the precision rate was 92.60%.^30^ Varsha et al. used a variety of machine learning classification techniques, including Random Forest (RF), Adaboost, XGBoost, Naïve Bayes (NB), Logistic Regression (LR), and Support Vector Machine, etc on a dataset of breast cancer patients.^31^ The techniques were evaluated using various performance measures. Among all models, it has been discovered that the decision tree and XGBoost classifier have the highest accuracy (97%). An additional study was to create a new neural network for breast cancer diagnosis, we integrated US characteristics extracted by a modified VGG‐11 network with pictures rebuilt from a DOT deep learning auto‐encoder‐based model, which was inspired by a fusion model deep learning technique.^32^ After training on simulation data and refining it with clinical data, the mixed neural network model attained an AUC of 0.931. Mahendran et al. proposed a machine‐learning model based on blood profile data to classify metastasis cancer.^33^ To categorize cancer metastases, Mahendran et al. presented a machine‐learning model based on blood profile data. An 83% accuracy rate with an AUC of 0.87 was demonstrated by a Decision Tree (DT) classifier. Next, to develop a web application for reliable MBC patient diagnosis, they implemented DT classifiers using Flask. Lastly according to Cengiz et al.,^34^ proposed a CNN‐based breast cancer classification technique from noisy breast histopathological images. Initially, the photos in the utilized data set were supplemented with various kinds and levels of noise. Subsequently, the Wavelet Transform (WT) technique was utilized to eliminate noise from photographs. The suggested framework classified breast cancer with 86% accuracy.

This article discusses computer‐assisted image processing of histopathology to diagnose the presence of IDC and metastatic cancer cells and for this, we used Kaggle's version of the PatchCamelyon dataset. The benchmark PatchCamelyon is a recent and demanding data collection for the classification of images. It comprises 327,680 color images of size 96 × 96 taken from lymph node segment histopathologic scans. PCam offers a modern standard for machine learning frameworks. The visual analysis of images refers to inconsistency in diagnosis after obtaining a digital histology image from a biopsy specimen. Computer‐assisted systems are used to resolve this problem that providing an objective assessment of diseases.^35^ The main novel aspects of this work are next summarized.

1. Novel Model: We present our customized unique CNN‐based model for both IDC and metastasis‐type breast cancer classification. By using simple patching and normalization prepossessing, we significantly improve accuracy compared with state‐of‐the‐art other research approaches even pre‐trained well‐known models.
2. Dataset Invariance: The proposed Branch CNN‐based model is evaluated with breast histopathology datasets containing IDC and Metastasis cancer images and it generated state‐of‐the‐art results. Therefore, the model is generalized and dataset invariant.
3. Performance: A comprehensive empirical assessment is performed by systematically manipulating the model configurations and hyperparameters of CNN. Finally, an effective model with higher accuracy (of 95% and 89% for Metastasis and IDC breast cancer respectively) is developed compared with both transfer‐based learning and unique contributions of state‐of‐the‐art machine learning approaches.

The remaining portion of the paper is divided into three sections. In Section 2, we discuss the methodology of our work which includes dataset collection, prepossessing, a brief discussion on the CNN model architecture, model parameters, layers, activation function, and optimization algorithm. The results of both IDC and metastasis‐type breast cancer classification are given in Section 3. Finally, Section 4 discusses the conclusion and future aspects of our research work.

## 2 METHODOLOGY

**FIGURE 1:**
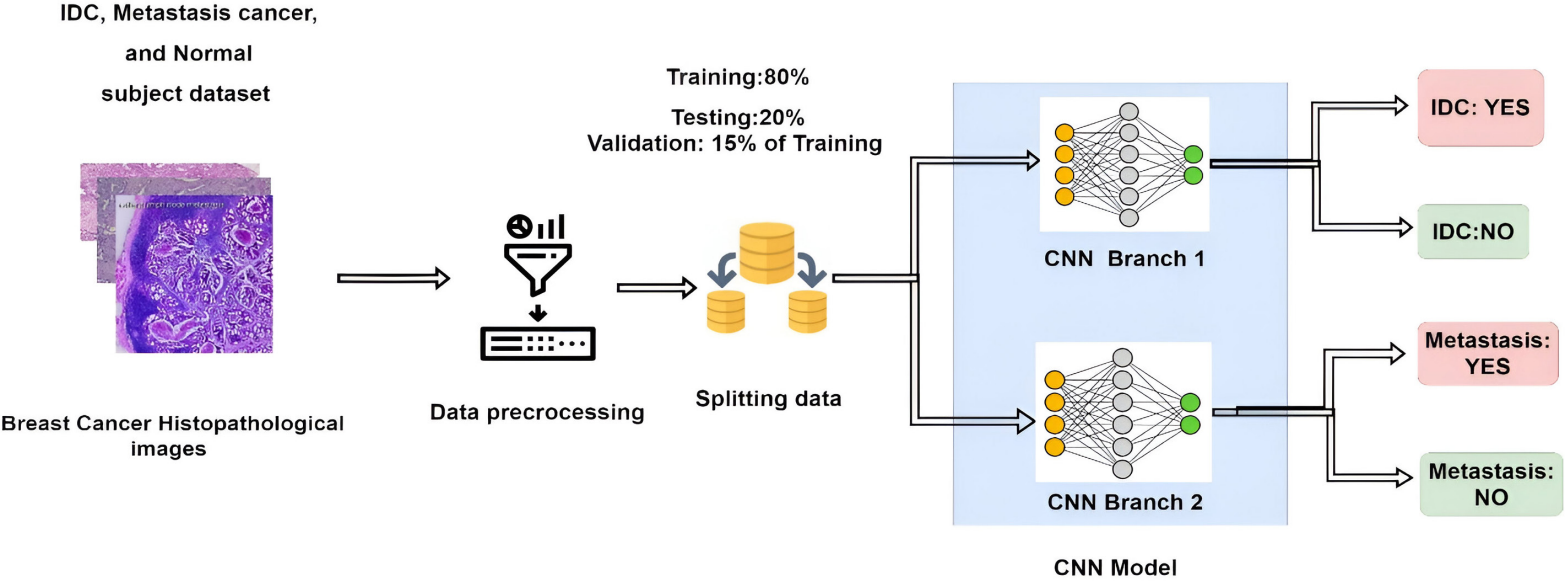
The overall framework of our proposed CNN‐based model to predict breast cancer status. Model inputs breast cancer‐based histopathological images are preprocessed, trained, and finally classified as IDC, metastasis, or none.

This section presents an overview of the used datasets, data preprocessing techniques, and model architecture, training of the model, activation function optimization algorithm. Our proposed branch CNN model takes breast histopathological images as input and predicts cancer type as output. The overall structure of our approach for classifying breast cancer is shown in Figure 1. The CNN model is comprised of two different branches for predicting the presence of IDC and metastasis.

### 2.1 Dataset collection and preprocessing

**FIGURE 2:**
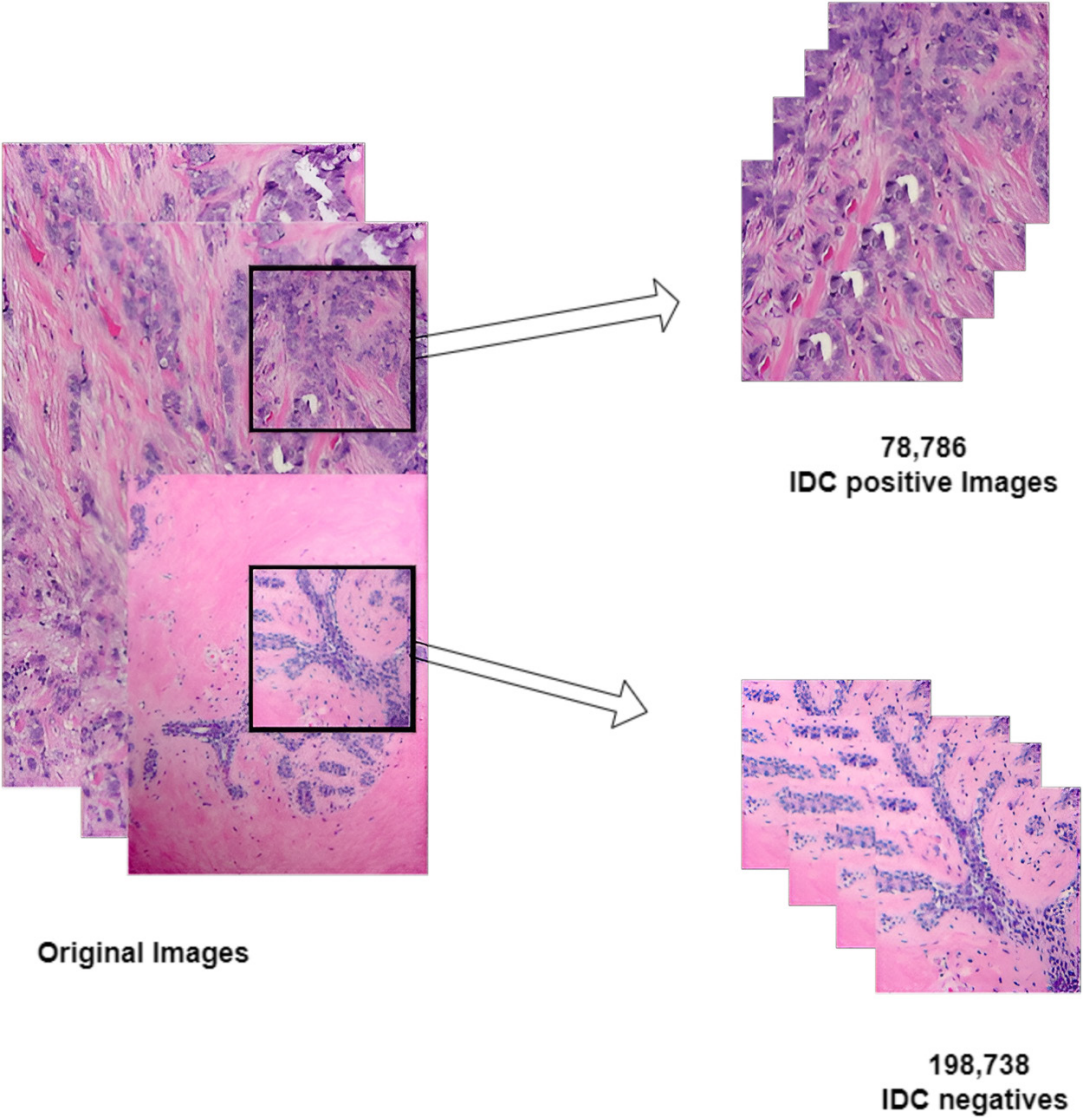
Represents patching of images.

As our comprehensive research on breast cancer classification deals with two types of binary classifications, we used a dataset for this evaluation process to meet the purpose. In the case of binary classification of IDC, a bundle of breast histopathology images has been used that is expansively available for the researchers as provided by the authors.^36^ The original dataset comprises BCa histopathologic slide images collected from 162 patients diagnosed with IDC at the Hospital of the University of Pennsylvania and The Cancer Institute of New Jersey. Evaluating the whole histopathology images is quite turbulent due to their large size, leading to the preprocessing of images as the size of the images was reduced to 277,524 total image patches that include 198,738 IDC negatives and 78,786 IDC positives. Figure 2 represents the parching process for our work. The format used for the classification purpose was viewing software ImageScope from Aperio, which was used to plot the annotations along with it. Regarding parameter examinations, the subsets of the used data comprise 84 training and 29 validations, while the remaining 49 were used for testing purposes. If our input image *I* is of size *H* × *W* (height *H* and width *W*), and we want to resize it to a new size ′*H*′ × *W*′. The resized image ′*I*′ is computed as follows:

For each pixel (*x*',*y*') in the resized image ′*I*′, where *x*' = 0,1, … *W*′ − 1 and *y*' = 0,1, … *H*′ − 1, compute the corresponding coordinates (*x*,*y*) in the original image *I* using:

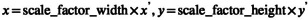




Then, we normalize our image before using it for training purposes. The mathematical notation for this type of normalization can be expressed as follows:

Given an input image I of size m × n with pixel values I_ij_ where i = 1,2…m and j = 1,2…n, the normalized image Inorm is calculated as:

(1) $$\;I_{\text{norm}}=\frac{I-\mu }{\sigma }$$

where, *μ* represents the mean of all pixel values in the image *I*. *σ* represents the standard deviation of all pixel values in the image *I*. Inorm represents the normalized image.

In the case of another binary detection regarding the benign and metastasis cancer cells, the PatchCamelyon benchmark dataset was used which is much more digitized and clinically modified for this metastasis detection purpose.^37^ This data cohort comprises 220 K training image files and 57.5 K files entitled based on the image identity. The train_labels.csv used here meets the purpose of serving the ground truth for images in the training location. Each positive indication represents that a minimum of one pixel of tumor tissues is present in the patch's 32‐by‐32‐pixel center.^38^, ^39^

### 2.2 Model architecture

In this section, we will discuss the overall architecture of our proposed branch CNN‐based breast cancer detection model. Model CNN layers, activation functions, and optimization algorithms will be discussed in this subsection.

#### 2.2.1 CNN layer

CNN is used primarily in computer vision as well as image processing tasks. It is designed to process and analyze grid‐like structured data such as images, audio, and video by using a set of convolutional layers to identify features and patterns in the data. In several computer vision tasks like semantic segmentation, object identification, and picture classification, CNNs have demonstrated exceptional outcomes, positioning them at the forefront of the field. One of the key benefits of a CNN‐based deep learning architecture is it needs preprocessing of the dataset or sometimes needs a very little bit of preprocessing. Its working principle is very similar to the human neuron and the learning process is very efficient compared to other deep learning models. The starting operation of a CNN model is just a convolution operation with filter and image.

In the CNN model, the filter is defined as the kernel. The convolutional layers play a crucial role in the architecture of CNN. In this layer, matrix multiplication is performed between the specific shape of the kernel and input images. In our proposed architecture, the main CNN layer contains two sub‐branches: CNN branch 1, and CNN branch 2. Each sub‐branch contains three sets of ReLU, convolution, and pooling layers. In the end, flattened and dense layers are connected. If we consider a single image output image after one convolution will be n_A_ × n_B_ × n_C_ based on the length of filters, input images, and weight. The bias is represented in expressions (2), (3), (4), and (5), respectively.

(2) $$\text{size}\_\text{of}\_\text{input}=n_{A}^{\left[l-1\right]}\times n_{B}^{\left[l-1\right]}\times n_{C}^{\left[l-1\right]}$$

(3) $$\text{size}\_\text{of}\_\text{filter}=f_{A}^{\left[l\right]}\times f_{B}^{\left[l\right]}\times f_{C}^{\left[l-1\right]}$$

(4) $$\text{size}\_\text{of}\_\text{weight}=\left(f_{A}^{\left[l\right]}\times f_{B}^{\left[l\right]}\times f_{C}^{\left[l-1\right]}\right)\times n_{C}^{\left[l\right]}$$

(5) $$\text{size}\_\text{of}\_\text{bais}=1\times 1\times 1\times n_{C}^{\left[l\right]}$$

The output layer at level l can be measured as by the equation (5) given below.

(6) $$n_{A}=\frac{n_{A}^{\left[l-1\right]}+2p^{\left[l\right]}-f^{\left[l\right]}}{S^{\left[l\right]}}+1$$

(7) $$\text{size}\_\text{of}\_\text{output}=n_{A}^{\left[l\right]}\times n_{B}^{\left[l\right]}\times n_{C}^{\left[l\right]}$$

The measurement of n_W_ is very similar to n_A_ shown in equation (6) where s^[l]^ and p^[l]^ represent the stride and padding size respectively. We used a 3 × 3 filter size in the two CNN branches in each convolution layer.

#### 2.2.2 Activation functions

Several activation functions can be used in CNNs. Some popular ones include ReLU, sigmoid, tanh, and softmax. In this research, the commonly used sigmoid activation function is applied. It squashes the input values within the range of zero to one. The mathematical expression for the sigmoid activation function can be expressed in equation (8).

(8) $$f\left(x\right)=\frac{1}{1+e^{\left(-x\right)}}$$

#### 2.2.3 Optimization algorithm

Optimization algorithms are used to update the weights of the neural network during training. Several optimization algorithms can be used for CNNs. Some of the most commonly used ones are stochastic gradient descent (SGD), Adam, Adagrad, RMSprop, Adadelta, and Nesterov accelerated gradient (NAG). Here, we use the ‘Adam’ algorithm which is a very popular optimization algorithm that combines the advantages of the Adagrad and RMSprop algorithms. It uses a running average of both the first and second moments of the gradients to update the weights. Image moment which is denoted by Mij, and calculated as:

(9) $$M_{\mathrm{ij}}=\sum_{x}\sum_{y}x^{i}y^{j}.I\left(x,y\right)$$

where *i* and *j* are non‐negative integers denoting the order of the moment. *x* and *y* represent the pixel coordinates within the image. *I*(*x*,*y*) is the intensity of the pixel at coordinates (*x*,*y*).

Since f(x,y) is a binary image, its value will always be one or zero. It is simple to observe that this equation assigns a value of one to each pixel in our image. In essence, it determines our binary image's area. By computing the core moment, we can determine the moments for every single blob. Our common formula is to determine the central moment of a blob about any point.

(10) $$\mu_{\mathrm{ij}}=\sum_{x}\sum_{y}{\left(x-\bar{x}\right)}^{i}{\left(y-\bar{y}\right)}^{j}.I\left(x,y\right)$$

where $\bar{x}$ and $\bar{y}$ are the centroid coordinates of the image, calculated as the mean of all pixel coordinates weighted by the intensity values. The Adaptive Moment Estimation (Adam) gradient descent algorithm is used to optimize the neural network. For ‘Adam’ the update rule of the parameter is like below. For each parameter wj; If we represent; η = Initial learning rate; gt = Gradient at time t along wj; Vt = Exponential average of the gradient along wj; St = Exponential average of the square of the gradient along wj; β1, β2 = Hyperparameters.

Then,

(11) $$\;V_{t}=\beta_{1}*V_{t-1}-\left(1-\beta_{1}\right)*g_{t}$$

(12) $$S_{t}=\beta_{2}*S_{t-1}-\left(1-\beta_{2}\right)*g_{t}^{2}$$

(13) $$\Delta w_{t}=-\eta \frac{V_{t}}{\sqrt{S_{t}+\epsilon }}*g_{t}$$

(14) $$\;w_{t+1}=w_{t+1}+\Delta w_{t}$$

Again, it can be written that,

(15) $$\theta_{t+1}\;=\;\theta_{t}\;-\;\frac{n}{\sqrt{\hat{v}}+\in }\;{\hat{m}}_{t}$$

(16) $${\hat{m}}_{t}\;=\;\frac{m_{t}}{1-\beta_{1}^{t}}$$

(17) $${\hat{v}}_{t}\;=\;\frac{v_{t}}{1-\beta_{2}^{t}}$$

where *m*_*t*_ and *v*_*t*_ indicate the estimates of the 1st moment (i.e. mean) and the second moment (i.e. un‐centered variance) of the gradient respectively.

Here for the ‘Adam’ optimizer the best fit value of decay rate *β*_1_ = 0.9, *β*_2_ = 0.999, and ϵ = 10^−8^.

#### 2.2.4 Training model

**FIGURE 3:**
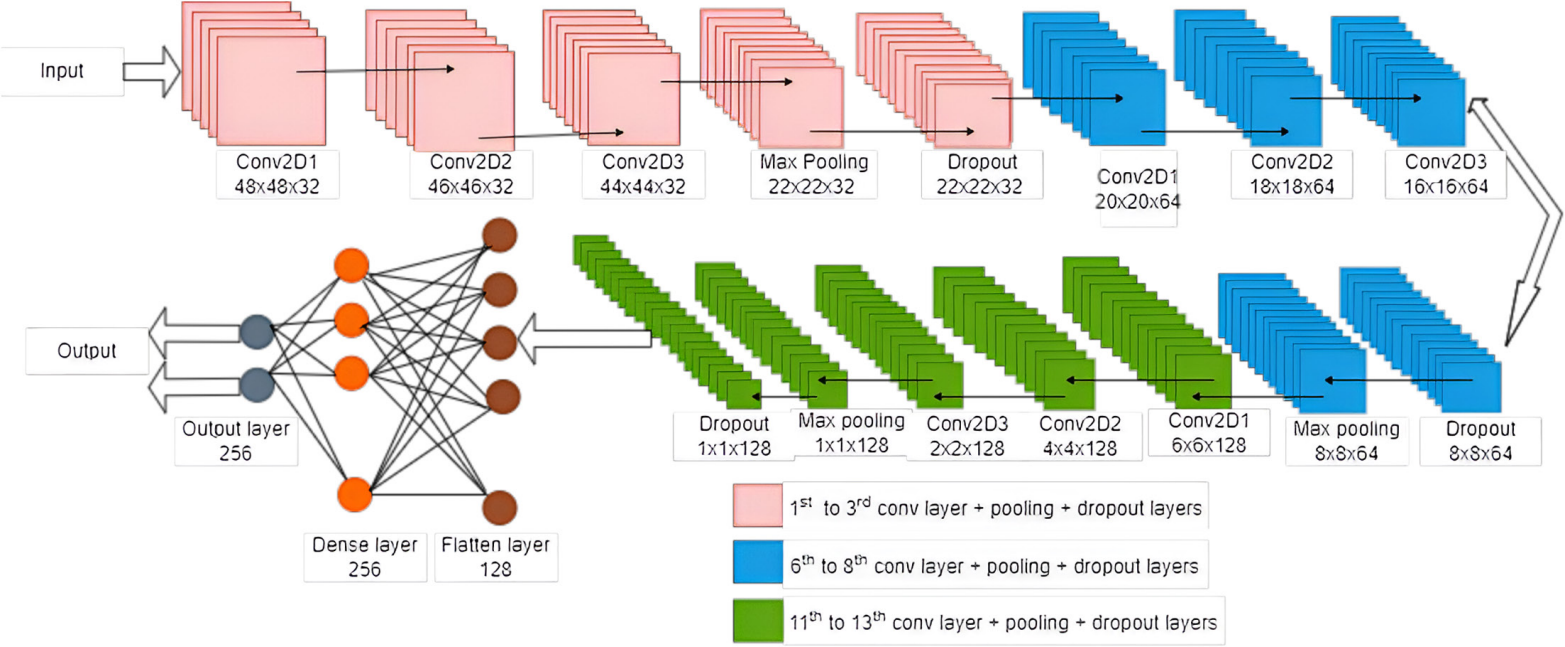
Framework of the proposed CNN branch 1 for IDC breast cancer classification.

**FIGURE 4:**
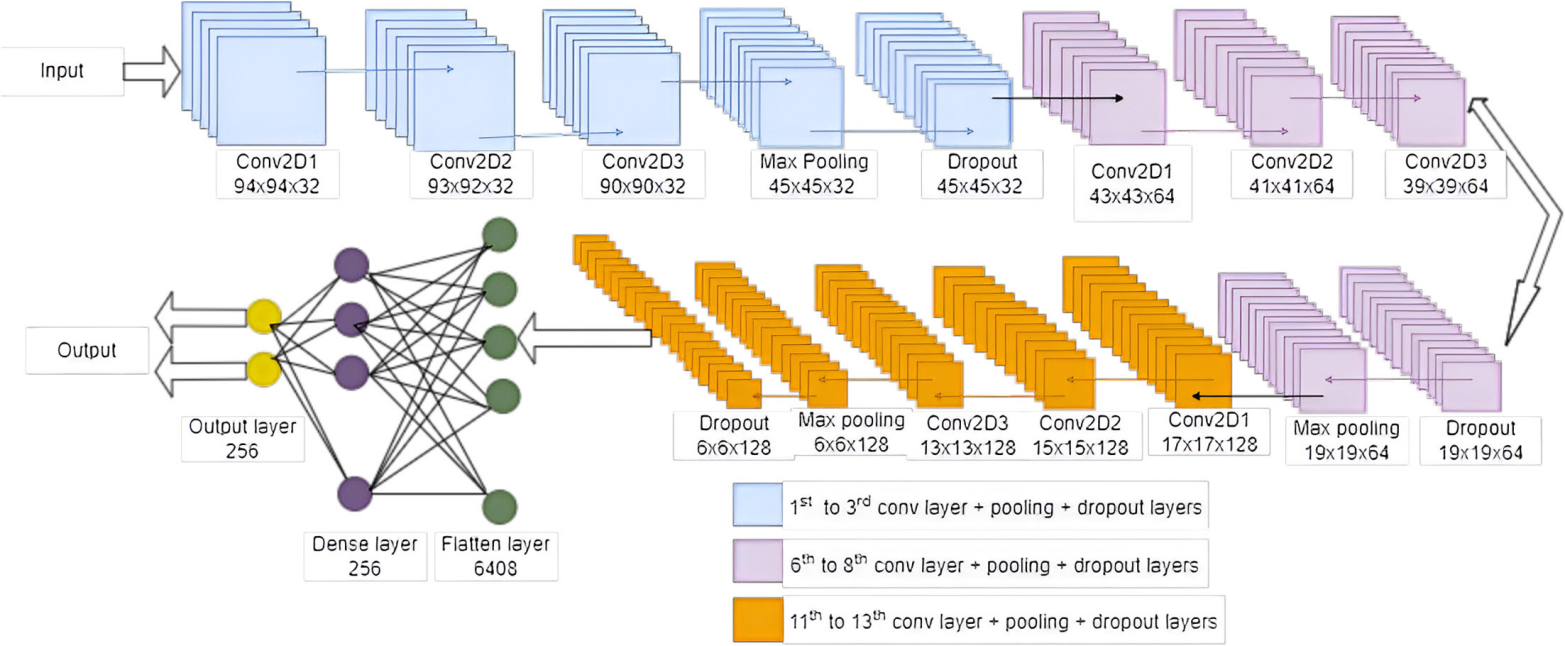
Framework of the proposed CNN branch 2 for metastasis breast cancer classification.

For both of the branches, we split our data for training and testing as 80% and 20%, respectively. We use 15% of our training data to validate our models. Figures 3 and 4 represent the subbranch layers with input and output dimensions. Here, we used *p* = 1 and *s* = 1 for the generalized value for overall the networks. The maximum polling approach is used in our network. To implement the learning process of our proposed model, Google Colab is employed. Table 1 is given to show the used parameters for our proposed model. The overall summary of the training process is shown in Tables 2 and 3.

**TABLE 1:** The values of our proposed CNN model hyperparameters.

Serial number	Hyperparameters	Values	Notation
1	Batch size	128	‐
2	Model dimension	128	$d_{\text{model}}$
3	Epochs for branch 1	40	‐
4	Epochs for branch 2	20	‐
5	Dropout rate	0.02	‐

**TABLE 2:** Summary of proposed model CNN branch 1.

Layer type	Output dimension	# Parameters
Conv2D	None, 48, 48, 32	896
Conv2D	46 × 46 × 32	9248
Conv2D	44 × 44 × 32	9248
MaxPooling2D	22 × 22 × 32	0
Dropout	22 × 22 × 32	0
Conv2D	20 × 20 × 64	18,496
Conv2D	18 × 18 × 64	36,928
Conv2D	16 × 16 × 64	36,928
MaxPooling2	8 × 8 × 64	0
Dropout	8 × 8 × 64	0
Conv2D	6 × 6 × 128	73,856
Conv2D	4 × 4 × 128	147,584
Conv2D	2 × 2 × 128	147,584
MaxPooling2	1 × 1 × 128	0
Dropout	1 × 1 × 128	0
Flatten	128	0
Dense	256	33,024
Dropout	256	0
Dense	2	514
Total parameters: 514,306 Trainable parameters: 514,306 Non‐trainable parameters: 0		

**TABLE 3:** Summary of proposed model CNN branch 2.

Layer type	Output dimension	# Parameters
Conv2D	94 × 94 × 32	896
Conv2D	92 × 92 × 32	9248
Conv2D	90 × 90 × 32	9248
MaxPooling2D	45 × 45 × 32	0
Dropout	45 × 45 × 32	0
Conv2D	43 × 43 × 64	18,496
Conv2D	41 × 41 × 64	36,928
Conv2D	39 × 39 × 64	36,928
MaxPooling2	19 × 19 × 64	0
Dropout	19 × 19 × 64	0
Conv2D	17 × 17 × 128	73,856
Conv2D	13 × 13 × 128	147,584
Conv2D	13 × 13 × 128	147,584
MaxPooling2	6 × 6 × 128	0
Dropout	6 × 6 × 128	0
Flatten	4608	0
Dense	256	1,179,904
Dropout	256	0
Dense	2	514
Total params: 1,661,186 Trainable params: 1,661,186 Non‐trainable params: 0		

## 3 RESULTS AND DISCUSSION

In our experiment, we classified breast cancers of the IDC and metastatic types using a CNN model based on branch CNN. We divide the results section into two subsections in this section: one for model performance, and the other for a comparison of the model with the state of art models for classifying breast cancer.

### 3.1 Model performance

In this subsection, we will analyze the brunch‐wise model performance of our proposed research work. Tables 4 and 5 respectively represent the confusion matrix for IDC and metastatic model with a total dataset of 22,201 and 16,000. Figures 6 and 9 show training and validation accuracy are relatively high concerning the corresponding loss for both models.

**TABLE 4:** Confusion matrix for IDC classification result.

Predicted condition	Condition negative	Condition positive
IDC absent	13,655	2234
IDC present	576	5736

**TABLE 5:** Confusion matrix for metastasis type breast cancer classification.

Predicted condition	Condition negative	Condition positive
Metastasis absent	7629	371
Metastasis present	491	7509

#### 3.1.1 For CNN branch 1

**FIGURE 5:**
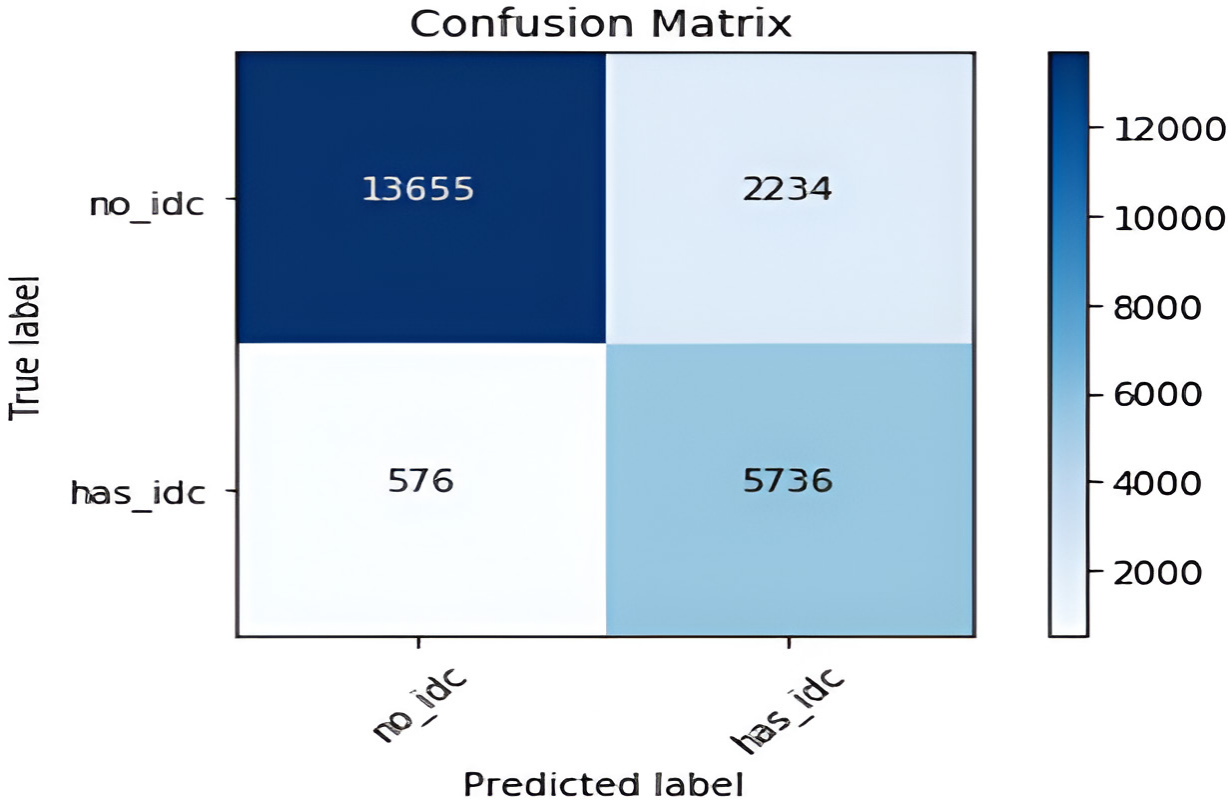
Confusion matrix of the proposed model for IDC.

Figure 5 and Table 4 depict the confusion matrix for IDC breast cancer. This represents the computational breast cancer hypothetical results compared with the actual data. The 5736 images predicted correctly the presence of IDC while 13,655 are identified as the absence of IDC; the machine anticipated result and the actual value are similar. Moreover, the false negative and false positive scores are 2234 and 576 respectively. This matrix is crucial for measuring the accuracy, precision, recall, and F1 score followed by the success rate of detection of IDC breast cancer.

**FIGURE 6:**
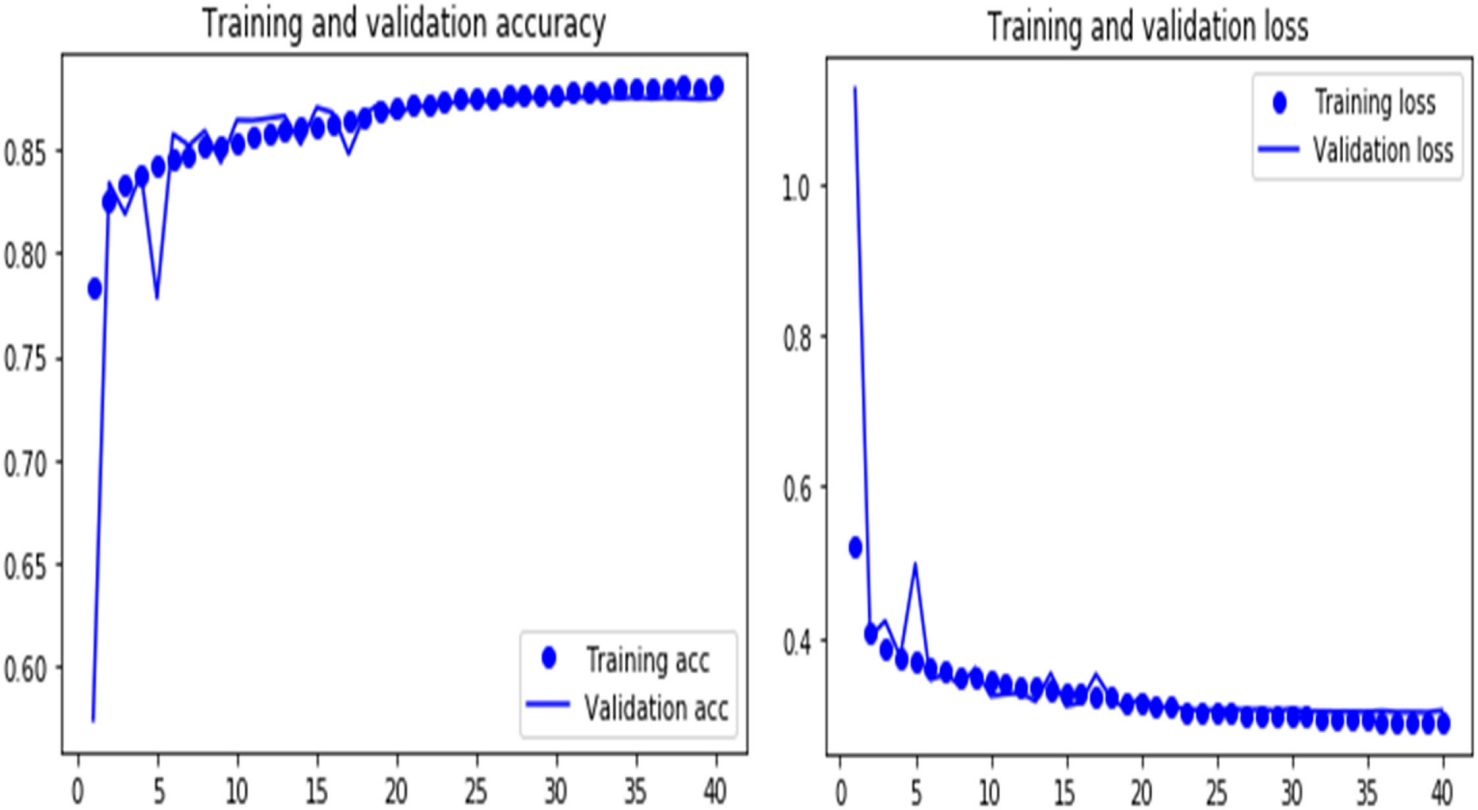
Training and validity accuracy and loss curves of our proposed model for IDC.

Figure 6 depicts the IDC breast cancer detection experiment, training accuracy, and validation accuracy both rose and stabilized at a certain point while training loss and validation loss both decreased. This denotes a model with the best fit—one that is neither overfit nor underfit.

#### 3.1.2 For the CNN branch 2

**FIGURE 7:**
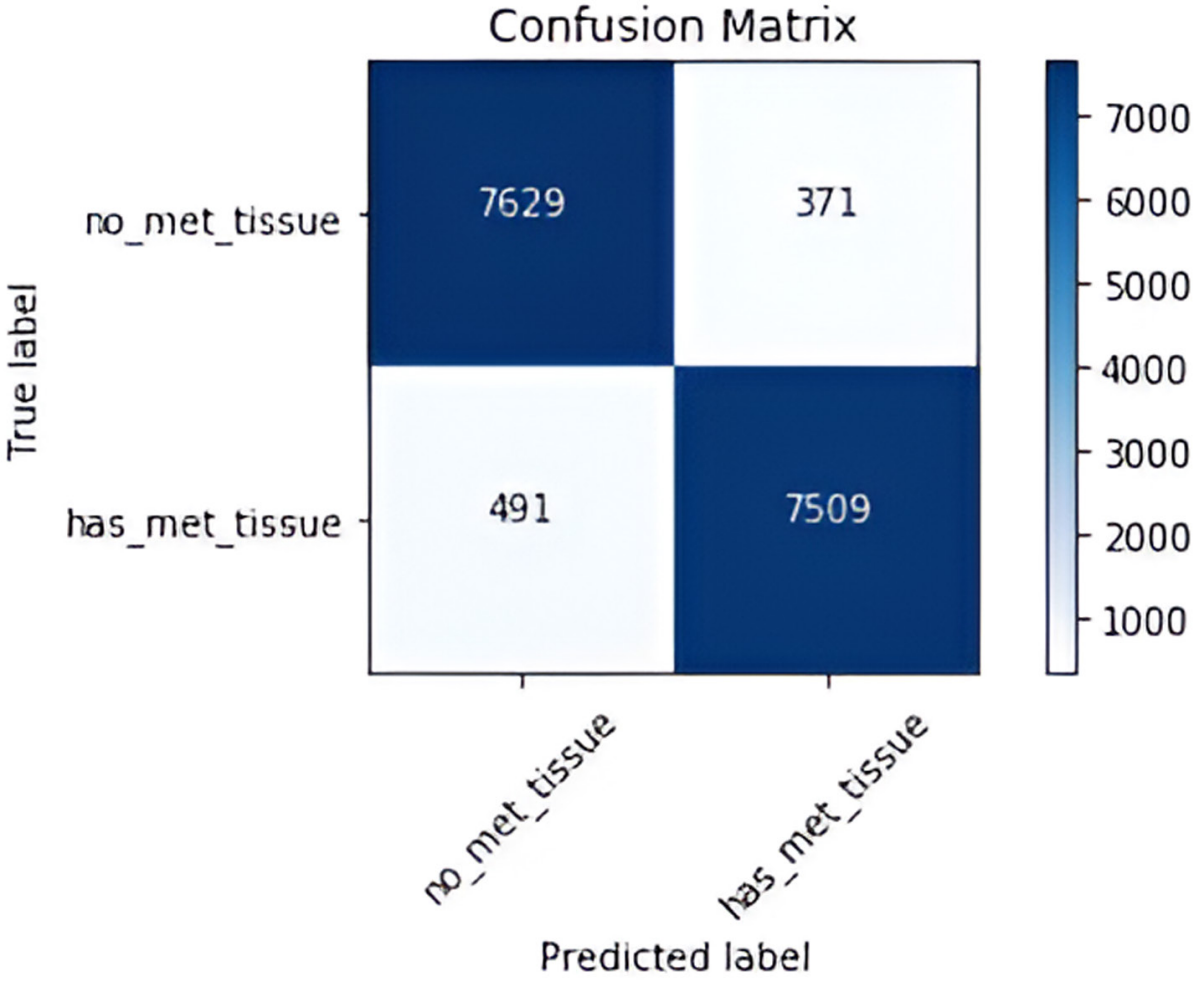
Confusion matrix of our proposed model for metastasis cancer classification.

Figure 7 and Table 5 elucidate a confusion matrix of metastasis breast cancer. This demonstrates the comparison between the theoretical computational findings for breast cancer and the real facts. The machine‐predicted result and the true value are comparable; the true positive value is 7509, and the true negative value is 7629. Additionally, the scores for false positive and negative are 491 and 371, respectively. This matrix is essential for analyzing the success rate of detecting breast cancer metastases as well as the accuracy, precision, recall, and F1 score.

**FIGURE 8:**
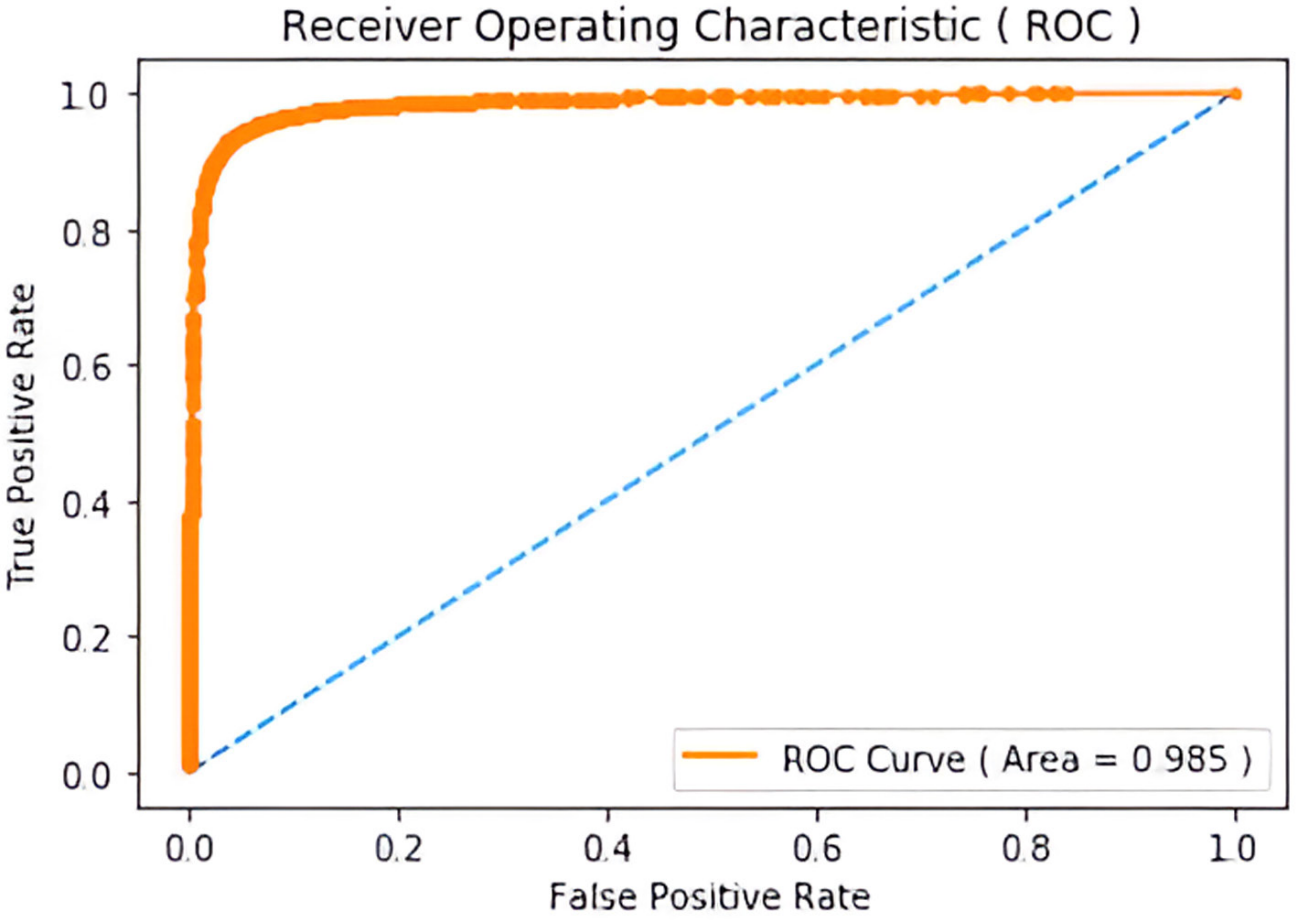
Receiver operating characteristic (ROC) of our proposed model for metastasis.

Figure 8 delineates the ROC curve for a CNN method used for metastasis breast cancer detection. By utilizing the ROC curve, the model's performance can be assessed in distinguishing between metastasis and non‐metastasis breast cancer cases. The true positive rate (sensitivity) represents the proportion of true positive cases (i.e., correctly identified metastasis cases) out of all metastasis cases, while the false positive rate (1‐specificity) represents the proportion of false positive cases (i.e., non‐metastasis cases incorrectly identified as metastasis) out of all non‐metastasis cases.

**FIGURE 9:**
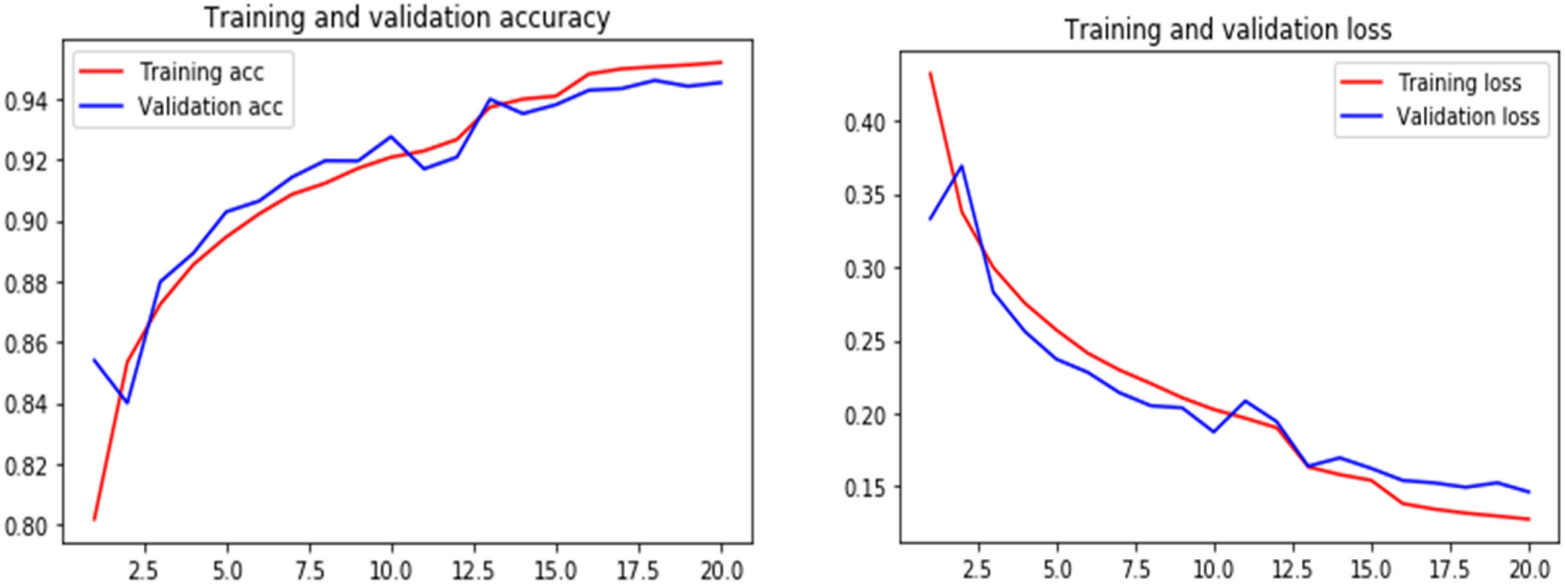
Training and validation accuracy and loss curves of our proposed model for metastasis cancer.

This graph indicates that the CNN method for detecting metastatic breast cancer has a high rate of correctly identifying positive cases (true positives), equal to 10, and a low rate of incorrectly identifying negative cases as positive (false positives), which is equal to nearly zero. Therefore, the ROC curve hugs the upper left corner of the graph. The area under the ROC curve (AUC) is a statistic that offers a comprehensive evaluation of the performance of the CNN method, with values of 0.985 which is closer to 1 indicating better performance. Figure 9 represents our training and validation accuracy and loss curve for both training and validation accuracy of our proposed metastasis‐type breast cancer detection. Here, Tables 6 and 7 represent the classification of different types of accuracy values for IDC and metastasis‐type breast cancer detection respectively.

**TABLE 6:** The classification report for IDC prediction (CNN branch 1).

Criteria	Precision	Recall	f1‐score	Support
IDC_absent	0.91	0.72	0.96	13,889
IDC_present	0.86	0.96	0.91	6312
Average/total	0.89	0.84	0.94	22,201

**TABLE 7:** All parameters contribute to predictive uncertainty for metastasis cancer.

Criteria	Precision	Recall	f1‐score	Support
Metastasis_absent	0.94	0.95	0.95	8000
Metastasis_present	0.95	0.94	0.95	8000
Average/total	0.95	0.95	0.95	16,000

**FIGURE 10:**
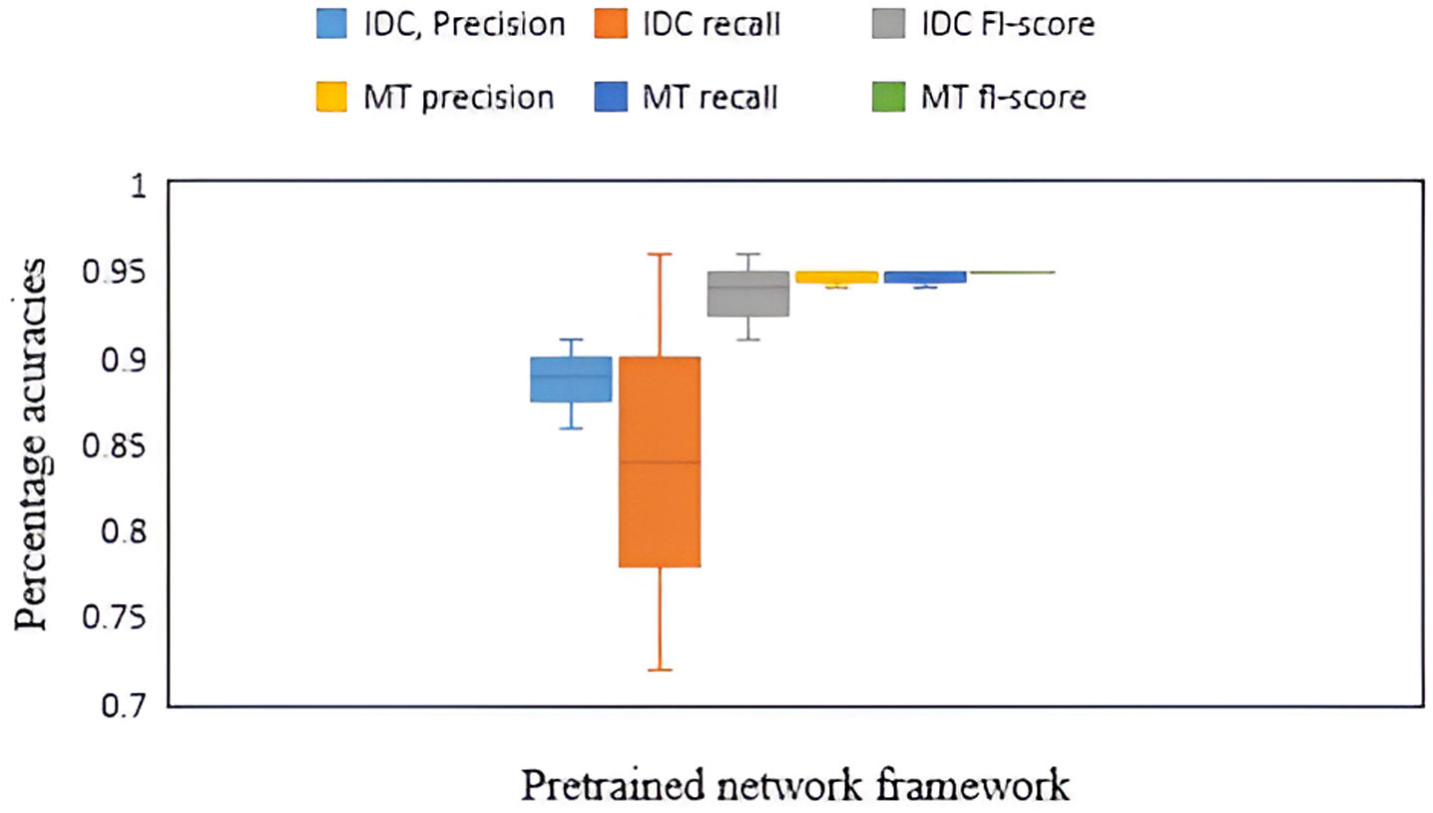
Box plot for predictive uncertainty.

The box plot, shown in Figure 10 depicts the level of accuracy of detecting IDC and metastasis breast cancer. The mean success rate of detection for metastasis cancer was 95% while the IDC rate was 89%. The F1 score depicts the average of precision and recalls in a harmonic manner that represents the highest level of accuracy for detecting metastasis tissue (MT) and IDC tissue respectively, comprising 95% and 94%. The precision was 89% for IDC prediction, on the other hand, the precision value for metastasis was greater, accounting for 95%. Moreover, the positive cases in the data (recall) were 84% for IDC tissue detection, meanwhile, the rate of accuracy for metastasis tissue was 95%.

### 3.2 Model comparison

We obtained an average of 89% accuracy for IDC models and 95% accuracy for the metastasis model, indicating that these models are more effective. For analysis, the proposed model is compared with state‐of‐the‐art models given in Table 8.

**TABLE 8:** Comparison of the proposed CNN models with other methods.

Authors	Method(s)	Cancer Types	Accuracy (%) ± SD
Kahya et al.^19^	Handcrafted features + classification	Metastasis	94.54
Zhongyi et al.^20^	CSDCNN + multi‐classification	IDC	93.2
Saxena et al.^21^	Pre‐trained network extracted traits + sSVM	IDC & Metastasis	90.12
Alanazi et al.^22^	CNN	IDC	87
Nguyen et al.^24^	Features concatenation network + transfer learning	IDC & Metastasis	92.63 ± 1.68
Ragab et al.^19^	DCNN‐SVM–AlexNet	Metastasis	87.2
Alom et al.^26^	IRRCNN	IDC	96.84
Gour et al.^27^	Transfer learning + ResHist CNN (based on ResNet‐152)	Metastasis	91.35 ± 2.3
Mehra et al.^28^	Fine‐tuned pre‐trained VGG16	Metastasis	92.60
Kamlesh Kumar et al.^39^	VGG16, InceptionV3	IDC	85.59, 82
S. Singh et al.^40^	ResNet‐50	IDC	85.21
Sara Hosseinzadeh Kassani et al^41^	DenseNet	Metastasis	83.10
Proposed Models	CNN	IDC & Metastasis	IDC 89 and Metastasis 95

A comparison of the accuracy rate of CNN base IDC and metastasis breast cancer prediction approach with the other machine learning base detection method is shown in Table 7. Kahya et al. presented a method for breast tumor classification using an adaptive sparse support vector machine that selects features, and they reported an average accuracy of 94.54% across all magnification factors for metastasis breast cancer detection. Zhongyi et al.,^22^ state the Complex Shifting‐Dilated Convolutional Neural Network (CSDCNN) multi‐classification method for the identification of IDC breast cancer. This approach obtained an average accuracy of 93.2% for classifying images with varying levels of magnification. Alanazi et al.^42^ utilized advanced deep CNN architectures in their deep learning methods with the BreaKHis database for predicting IDC breast cancer, achieving an accuracy rate of approximately 87% while requiring high‐end hardware resources and considerable training time. Moreover, Gour et al.^43^ trained transfer learning—ResNet‐152 networks with various input sizes for feature extraction from enhanced and preprocessed histopathology images. This process was able to detect the metastasis of breast cancer 91.35 ± 2.3 percent correctly. All of these models need high‐level preprocessing and long training time. Patches of size 32 × 32 and 64 × 64 were used to solve this problem and augment the training data, which were extracted from the Cancer Institute of New Jersey as well as the University of Pennsylvania biopsy images. Here, our CNN model needs very low‐level preprocessing however patch results were image level accuracy of an average of 89% accuracy for IDC models and 95% accuracy for the metastasis model, indicating that our model is more effective than the state‐of‐art models. We can assist physicians in detecting breast cancer levels by using our model in real‐time medical diagnosis applications.

## 4 CONCLUSIONS

In this study, we aimed to automatically classify the two main IDC and metastatic breast cancer. Here, two types of customized CNN models are proposed for the prediction of the cancer types separately along with comprising some state‐of‐art detection procedures and classification accuracy. We utilized microscopic histopathological images from the Cancer Institute of New Jersey and the University of Pennsylvania to train our models. CNN branches 1 and 2 were customized to detect IDC and metastasis breast cancer with an accuracy of 89% and 95%, respectively. We compared our classification accuracy with some state‐of‐the‐art models and found our proposed model to be functional in predicting breast cancer. In our future work, we integrate eXplainable Artificial Intelligence (XAI) techniques like Grad‐CAM, and SHAP with our proposed model to enhance its interpretability. So, oncologists can use our model to identify breast cancer with its current level, IDC, or metastasis. The future research plan includes proposing a more robust and generalized model utilizing a privately collected dataset along with a private one.

## AUTHOR CONTRIBUTIONS

**Tobibul Islam:** Conceptualization (equal); formal analysis (equal); investigation (equal); writing – original draft (equal). **Md Enamul Hoque:** Conceptualization (equal); supervision (equal); writing – review and editing (equal). **Mohammad Ullah:** Formal analysis (supporting); investigation (equal); validation (supporting); writing – original draft (equal). **Toufiqul Islam:** Formal analysis (equal); investigation (equal); writing – original draft (equal). **Nabila Akter Nishu:** Data curation (equal); formal analysis (equal); investigation (equal). **Rabiul Islam:** Conceptualization (equal); methodology (supporting); writing – original draft (equal); writing – review and editing (equal).

## CONFLICT OF INTEREST STATEMENT

The authors have no conflict of interest.

## ETHICS STATEMENT

N/A as this research does not involve any human and/or animal procedure.

## Data Availability

This research data comes from the BCa histopathologic slide images of the University of Pennsylvania and the Cancer Institute of New Jersey, and the PatchCamelyon benchmark dataset. These are publicly available datasets, which can be found on the following weblinks. https://www.kaggle.com/datasets/andrewmvd/metastatic‐tissue‐classification, https://github.com/DataSystemsGroupUT/DC‐classification.
